# Reduced retinal microvascular density in patients with mixed connective tissue disease: an exploratory pilot study on the interplay between aging, renal function, and complement system

**DOI:** 10.3389/fimmu.2026.1724780

**Published:** 2026-02-05

**Authors:** Paola Triggianese, Eugenio Capparelli, Arianna D’Antonio, Carolina Nesi, Marco Lombardo, Barbara Kroegler, Alberto Bergamini, Raffaele Mancino, Anna Paola Mitterhofer, David Della-Morte, Carlo Nucci, Massimo Cesareo, Alessio Martucci

**Affiliations:** 1 Geriatric and Nutrition Unit, Department of Biomedicine and Prevention, Tor Vergata University, Rome, Italy; 2 Rheumatology Unit, Department of Systems Medicine, University of Rome Tor Vergata, Rome, Italy; 3 Ophthalmology Unit, Department of Experimental Medicine, University of Rome Tor Vergata, Rome, Italy; 4 Nephrology and Dialysis Unit, Department of Systems Medicine, University of Rome Tor Vergata, Rome, Italy

**Keywords:** aging, complement system, mixed connective tissue disease, nailfold videocapillaroscopy, renal function, retinal vasculature, optical coherence tomography angiography (OCT-A)

## Abstract

**Background:**

Mixed connective tissue disease (MCTD) is a systemic autoimmune disease with overlapping features with systemic lupus erythematosus, systemic sclerosis, and inflammatory idiopathic myopathies, characterized by anti-U1-RNP antibodies. Although subclinical retinal microvascular changes have been described in other connective tissue diseases, such data are lacking in patients with MCTD.

**Methods:**

We performed a cross-sectional exploratory pilot study including patients with a defined diagnosis of MCTD according to one of the sets of classification criteria and age- and sex-matched healthy controls (HC), with elderly individuals equally distributed. Data on disease duration, renal function (creatinine and eGFR), and complement levels (C3 and C4) were recorded. All participants underwent optical coherence tomography angiography (OCT-A) to evaluate retinal vessel density (VD) using parafoveal, perifoveal, and foveal scans, whole images, and foveal avascular zones (FAZs). Nailfold videocapillaroscopy (NVC) was performed in patients with MCTD on four fingers of both hands to assess microangiopathy patterns.

**Results:**

Patients with MCTD (n = 20, mean age 60.6 ± 11.4 years, 85% females) showed a significant reduction in both superficial and deep retinal VD across all evaluated regions compared with 20 HC. In patients with MCTD, deep retinal VD was inversely correlated with disease duration (r = −0.6, P = 0.0003) and directly correlated with eGFR (r = 0.4, P = 0.05). In patients with MCTD, C3 levels were positively correlated with age (r = 0.4, P = 0.03) and superficial parafoveal VD (r = 0.5, P = 0.008). In MCTD, NVC abnormalities, including non-specific microangiopathy and scleroderma patterns, occurred in 60% (n = 12) of cases and did not correlated with OCT-A findings.

**Conclusion:**

Patients with MCTD present subclinical retinal microvascular abnormalities detectable by OCT-A. Our hypothesis-generating study suggests that retinal vascular changes may be linked to disease duration, renal function, and complement levels. OCT-A may be a useful tool for assessing MCTD.

## 1 Introduction

Mixed connective tissue disease (MCTD) was first described in 1972 and is recognized as a disease syndrome with overlapping features of systemic sclerosis (SSc), systemic lupus erythematosus (SLE), and polymyositis/dermatomyositis (PM/DM), associated with antibodies to RNAse-sensitive extractable nuclear antigen (ENA) (1). The main clinical findings in MCTD are swollen hands and fingers, myositis, polyarthritis/-arthralgia along with polyserositis, abnormalities in lung and esophageal function, and lymphadenopathy (1, 2). As in other connective tissue diseases (CTDs), early in the disease course, patients may have nonspecific symptoms, including migraine and Raynaud’s phenomenon (RP), often leading to a misdiagnosis and/or a diagnosis of undifferentiated connective tissue disease (UCTD) (2). However, MCTD can also represent an early stage of specific systemic CTDs, such as SSc and SLE, or defined overlap syndromes (OS) (3). Cappelli et al. described that typical clinical SSc or SLE features can be documented more frequently during disease progression in the same patient, along with specific autoantibodies associated with the evolution into defined SLE or SSc (4). Nevertheless, in MCTD, distinct autoantibodies (anti-U1-snRNP) persist throughout the disease course (4). However, although MCTD has been considered a subtype of other defined CTDs, it has been appropriately recognized as an independent entity because of the specific pattern of organ involvement and its characteristic association with pulmonary arterial hypertension (PAH), aseptic meningitis, and trigeminal neuropathy (5). MCTD is also associated with interstitial lung disease (ILD), which usually occurs as non-specific interstitial pneumonia (NSIP), usual interstitial pneumonia (UIP), and ground glass opacity (GGO) on high-resolution computed tomography (HRCT) scans (6). Authors described that ILD in MCTD might be associated with microvascular changes at nailfold videocapillaroscopy (NVC) suggesting a role for NVC as a potential tool for early ILD detection (7, 8). Furthermore, recent evidence has documented specific correlations between NVC patterns and organ involvement, mainly skin thickening and ILD, in MCTD (7, 8).

As documented in the literature, microvascular abnormalities at the retinal level, particularly in the pre-symptomatic phase, have been described in patients with SLE and SSc (9, 10). Indeed, evidence from previous studies suggests a relevant role for optical coherence tomography angiography (OCT-A) as a non-invasive diagnostic tool for the early detection of subclinical retinopathy in systemic autoimmune diseases (8–11).

Therefore, starting from the assumption that MCTD has overlapping features with other CTDs, especially SLE and SSc, the aim of the present study was to investigate for the first time whether patients with MCTD show subclinical microvascular damage at the retinal level.

We conducted a cross-sectional exploratory and hypothesis-generating study aimed at analyzing for the first time the potential presence of subclinical microangiopathy at the retinal level from a highly selective cohort of patients with MCTD using OCT-A, a noninvasive imaging technique capable of visualizing the retinal microvasculature by detecting blood flow without intravenous dye injection (12). We also included NVC analyses to explore the potential correlations between NVC patterns and OCT-A findings (7, 8). Correlations between OCT-A and clinical phenotypes, as well as biochemical parameters, were analyzed in the MCTD cohort.

## 2 Methods

A single-center cross-sectional comparative study with a control group included, from December 2023 to December 2024, patients with a defined diagnosis of MCTD recruited from the Tertiary Care Center of the Rheumatology Unit, Tor Vergata University Hospital in Rome (Italy). Healthy controls (HC) were enrolled from subjects referred to the Ophthalmology Unit at the same hospital. Inclusion criteria were: (1) a defined diagnosis of MCTD according to at least one of the following sets of classification criteria: Sharp’s, Kasukawa, Alarcón-Segovia, or Kahn’s (13); (2) age ≥18 and ≤80 years; (3) best-corrected visual acuity (BCVA) ≤0.5 logMAR; (4) intraocular pressure (IOP) <21 mmHg on diurnal testing with measurements using Goldmann applanation tonometry; (5) spherical equivalent refractive error between −6.0 and +4.0 diopters (10). The exclusion criteria were as follows: (1) established primary ocular diseases, including glaucoma; (2) chronic kidney disease (CKD) with abnormal renal function (defined as eGFR <45 mL/min/1.73 m²); (3) systemic disorders with known retinal involvement, such as diabetes and other autoimmune systemic diseases (current and past medical history); (4) pregnancy or lactation; (5) neoplasia; and (6) systemic treatments affecting retinal function (8).

Among 28 consecutive patients with MCTD, 20 fulfilled the inclusion criteria and completed the study. They were compared with 20 age- and sex-matched HC. Demographic and clinical data were collected. The records included disease duration and age at disease onset/diagnosis, concomitant disorders, and therapies. At the study visit, none of the patients had been off all therapies for more than 1 year during follow-up. Serum levels of complement components (C3 and C4) and anti-nuclear antibodies (ANA) were obtained from all patients in the study. C3 and C4 levels were measured using nephelometric assays (normal values 90 mg/dL–180 mg/dL and 10 mg/dL–40 mg/dL for C3 and C4, respectively), while ANA detection was conducted using IFA performed with HEp-2 cells (positive at the 1:160 dilution). Concomitant ENA (anti-CENP-A/-B, anti-Scl-70, anti-SSB/La, anti-Jo-1, anti-U1-RNP, anti-Ro52, and anti-Ro60) were also screened and confirmed by chemiluminescence assays. Serum glucose and creatinine levels were added to the panel to confirm glycemic homeostasis and renal function, respectively, with normal values were of 70 mg/dL–99 mg/dL for glucose and 0.7 mg/dL–1.2 mg/dL for creatinine. The estimated glomerular filtration rate (eGFR, according to the 2012 Chronic Kidney Disease Epidemiology Collaboration equation, expressed in mL/min/1.73 m^2^) and urine analysis (including pH, blood, protein, glucose, ketone, urobilinogen, and nitrite) were added.

MCTD disease assessment was conducted by expert immunologists/rheumatologists in accordance with good clinical practice including specialized visits and a tailored organ involvement diagnostic work-up as needed (e.g., pneumologist, nephrologist) (14).

All participants included in the study underwent ophthalmological evaluation at the Ophthalmology Unit of the Tor Vergata University Hospital in Rome (Italy). BCVA was measured using a standard LogMAR eye chart (15). IOP was determined using Goldmann applanation tonometry (16). Both eyes of each participant were examined with a 6 mm × 6 mm scanning protocol of the macular area using the Avanti Angiovue OCT-A (Optovue XR Avanti, Fremont, CA, USA), Software RTVue XR v.2018.1.1.63. The instrument’s built-in software provided the values of structural measures (foveal and parafoveal thickness, FT and PFT) and vessel density (VD) of the superficial and deep whole image (SWD, DWD), superficial and deep foveal density (SFD, DFD), superficial and deep parafoveal density (SPFD, DPFD), and superficial and deep perifoveal density (SPeFD, DPeFD). The foveal avascular zone (FAZ) areas were also measured (8). As quality controls, low-quality index (<7), presence of blink artifacts, motion or doubling artifacts caused by poor fixation, and media opacities obscuring the view of the vasculature represented criteria for poor image quality, and thus exclusion criteria (17, 18). OCT-A measurements were performed at the same time of the day for all the participants in the study.

The control group consisted of 20 HC who were age/sex and refractive index/BCVA matched with MCTD patients. Analyses evaluated both eyes from all the participants since, as reported in the literature, an appropriate and accepted approach is to use the mean value of both eyes for each participant, thereby ensuring independence at the subject level. Accordingly, analyses based on the average binocular data were considered methodologically consistent.

Patients with MCTD also underwent NVC performed by expert immunologists/rheumatologists, blinded to OCT-A findings, in accordance with good clinical practice, using NVC (Inspectis Digital Capillaroscope Light CAP-1). NVC was performed on four fingers of both hands, and NVC patterns were divided into non-specific microangiopathy and scleroderma-like patterns (7). Non-specific microangiopathic abnormalities comprised enlarged capillary, giant capillary, microhemorrhage, tortuous capillary, branched capillary, capillary disorganization or loss, neoangiogenesis, edema, and sub-papillary venous plexus; flow was also evaluated (19). Scleroderma-like patterns included early, active, and late patterns, as described by Cutolo et al. (20). All patients with MCTD in the study underwent lung HRCT scans, according to good clinical practice, at the time of the study. Based on the collected observations, concomitant ILD was defined as UIP, NSIP, or GGO (21). In addition, suspected PAH had already been screened in all the included MCTD patients using two-dimensional transthoracic echocardiography at rest, according to recommendations (22).

The study has been carried out according to the code of Ethics of the World Medical Association (Declaration of Helsinki) for experiments involving humans (updated 2013). Informed consent was obtained from all subjects, and the study was approved by the scientific ethic committee of the Tor Vergata University Hospital in Rome (Italy).

### 2.1 Statistical analysis

The D’Agostino and Pearson omnibus tests were used to assess data normality. Mean and standard deviation (SD) were used to express normally distributed variables, while non-normally distributed variables were analyzed using the median and percentile ranges. The parametric unpaired T test or the nonparametric Mann–Whitney U test was used to compare continuous variables, when appropriate. Percentages and absolute frequencies were used for categorical variables, which were compared using the Chi-squared test or Fisher’s exact test, when appropriate. The significance of any correlation was determined using Pearson’s correlation test. Considering the prevalence of MCTD, which classifies it as a rare disease, the final sample size, although small, reflects the maximum number of patients who met the inclusion criteria and completed the study at a single reference center for the disease. However, as the study was designed as an exploratory pilot study with hypothesis-generating aims, the relatively small size of the MCTD cohort could lead to a large effect size in the multiple regression analysis.

Statistical significance was set at P <0.05. All statistical analyses were performed using GraphPad Prism v. 10 (GraphPad Software).

## 3 Results

### 3.1 Study population

The MCTD cohort included 20 patients (**Table 1**) who were compared with 20 age (56.8 ± 10 y.o.), and sex (F 13/20, 65%) matched HC. Elderly patients (≥65 y.o.) were present at a similar prevalence in the MCTD (6/20, 30%) and HC (7/20, 35%) groups. Anti-U1-RNP antibodies were detected in 100% of the patients. Most patients were ANA-positive, while other ENA occurred in 20% (n = 4) of cases. RP was registered in the entire study population. Joint involvement was the main clinical finding, followed by ILD (**Table 1**). At the time of the study, eight (40%) patients were on steroids with a median dosage of 8.75 mg/day (range, 5 mg/day–10 mg/day) of prednisone or equivalent (**Table 2**).

**Table 1:** Data from the study population.

Patient's main clinical and demographic characteristics	MCTD (n = 20)
Age at the study (yrs)	60.6 ± 11.4
Females (N/%)	17/85
Elderly (N/%)	6/30
Age at the symptom’s onset (yrs)	48.7 ± 12.9
Age at the diagnosis (yrs)	52.2 ± 13.3
Disease duration (yrs)	9.7 ± 7.0
Clinical features	
Raynaud phenomenon (N/%)	20/100
Arthritis/Arthralgia (N/%)	15/75
TEE-PAH (N/%)	10/50
ILD (N/%)	14/60
Gut (N/%)	7/35
Digital ulcers (N/%)	7/35
Myositis/Myalgia (N/%)	6/30
NS including trigeminal neuralgia (N/%)	2/10
Laboratory assays	
C3 (mg/dl)	104.5 ± 28.3
C4 (mg/dl)	20.5 ± 7.9
ANA ≥1:160 (N/%)	13/65
U1-RNP positivity (N/%)	20/100
Other ENA (N/%)	4/20
glucose (mg/dl)	80.7 ± 17.3
creatinine (mg/dl)	0.77 ± 0.3
eGFR (mL/min/1.73 m²)	90.9 ± 39.6
Clinical pattern	
Predominantly SSc (N/%)	9/45
Predominantly SLE (N/%)	7/35
Predominantly IIM (N/%)	4/20

MCTD, mixed connective tissue disease; TEE, transthoracic echocardiogram; PAH, pulmonary arterial hypertension as suspected PAH; ILD, interstitial lung disease; NS, nervous system; ANA, anti-nuclear antibodies; C3/C4, complement; SSc, systemic sclerosis; SLE, systemic lupus erythematosus; IIM, idiopathic inflammatory myopathies. Continuous variables are shown as means ± SD, while categorical variables as absolute frequencies (N) and percentages.

**Table 2:** Treatments in the study population.

Treatment	MCTD (n = 20)
PDN/PDN-equivalent (N/%)	8/40
PDN/PDN-equivalent daily dose (mg/dl)	8.75 (5–10)
Hydroxychloroquine (N/%)	15/75
Mycophenolate (N/%)	5/25
Methotrexate (N/%)	4/20
Azathioprine (N/%)	2/10
Cyclosporine (N/%)	1/5
Rituximab (N/%)	3/15
Nintedanib (N/%)	2/10

MCTD, mixed connective tissue disease; PDN, prednisone; continuous variables are shown as median ± IQR, while categorical variables as absolute frequencies (N) and percentages.

A total of 38 eyes of patients with MCTD were included in the study. Both BCVA and IOP values were within the normal range in MCTD patients (BCVA 0.0 logMar, IOP 15.4 ± 1.37 mmHg) as in HC (BCVA 0.0 logMar, IOP 16 ± 1.3 mmHg).

### 3.2 Retinal vessel density by OCTA

Retinal VD was significantly decreased in patients with MCTD compared to that in HC in SWD, SFD, and SPFD scans (P <0.0001 for all comparisons) (**Table 3**). Furthermore, deep vascular plexi were significantly reduced in patients with MCTD than in HC in the DWD and DPFD scans (P <0.0001 for both comparisons) (**Table 3**). Moreover, perifoveal density was significantly decreased in patients with MCTD compared to that in HC in both deep (P <0.01) and superficial (P <0.001) vascular scans (**Table 3**). There were no differences in the FAZ areas between the MCTD patients and HC (**Table 3**). In addition, OCT scans of retinal thickness were similar between patients and HC (**Table 4**).

**Table 3:** Retinal vessel density by optical coherence tomography angiography (OCT-A).

OCT-A	MCTD (n = 38)	HC (n = 40)	*P*
SWD mean ± SD	48.6 ± 4.3	52.5 ± 3.1	<0.0001
SWD min–max	36.9–55.2	45–57	*ns*
SPFD mean ± SD	48.5 ± 9.5	55.2 ± 3.2	<0.0001
SPFD min–max	13.3–58.2	47.4–60.1	*ns*
SFD mean ± SD	20.9 ± 7.1	31.6 ± 8.9	<0.0001
SFD min–max	10–39.1	14.8–44.6	*ns*
DWD mean ± SD	50 ± 7.7	58.5 ± 3.4	<0.0001
DWD min–max	34–60.2	47.7–64.2	*ns*
DPFD mean ± SD	54.5 ± 5.8	61.2 ± 3.2	<0.0001
DPFD min–max	41.7–64	51.2–66.6	*ns*
DFD mean ± SD	36.8 ± 6.6	36.6 ± 4.6	*ns*
DFD min–max	22.9–48.4	26.3–45.3	*ns*
DPeFD mean ± SD	52 ± 7.7	57.7 ± 6.4	<0.01
DPeFD min–max	33.8–67.8	43.8–66.8	*ns*
SPeFD mean ± SD	51.2 ± 5.2	59.3 ± 3.6	<0.001
SPeFD min–max	42.8–66.8	52.2–65	*ns*
FAZ mean ± SD	0.3 ± 0.2	0.2 ± 0.1	*ns*

OCT-A, optical coherence tomography angiography; MCTD, mixed connective tissue disease; HC, healthy controls; SWD, superficial whole density; SPFD, superficial parafoveal density; SFD, superficial foveal density; DWD, deep whole density; DPFD, deep parafoveal density; DFD, deep foveal density; DPeFD, deep perifoveal density; SPeFD, superficial perifoveal density; FAZ, foveal avascular zone. Continuous variables are shown as means ± SD and range min–max.

**Table 4:** Retinal thickness by optical coherence tomography (OCT) scans.

Macular thickness values	MCTD (n = 38)	HC (n = 40)	*P*
FT (μm) mean ± SD	259.7 ± 22.1	262.5 ± 19	*ns*
FT (μm) min–max	216–318	220–297	*ns*
PFT (μm) mean ± SD	314.2 ± 20.5	324 ± 13.7	*ns*
PFT (μm) min–max	251–337	296–354	*ns*

MCTD, mixed connective tissue disease; HC, healthy controls; FT: foveal thickness; PFT: parafoveal thickness. Continuous variables are shown as means ± SD and range min–max.

**Figure 1:**
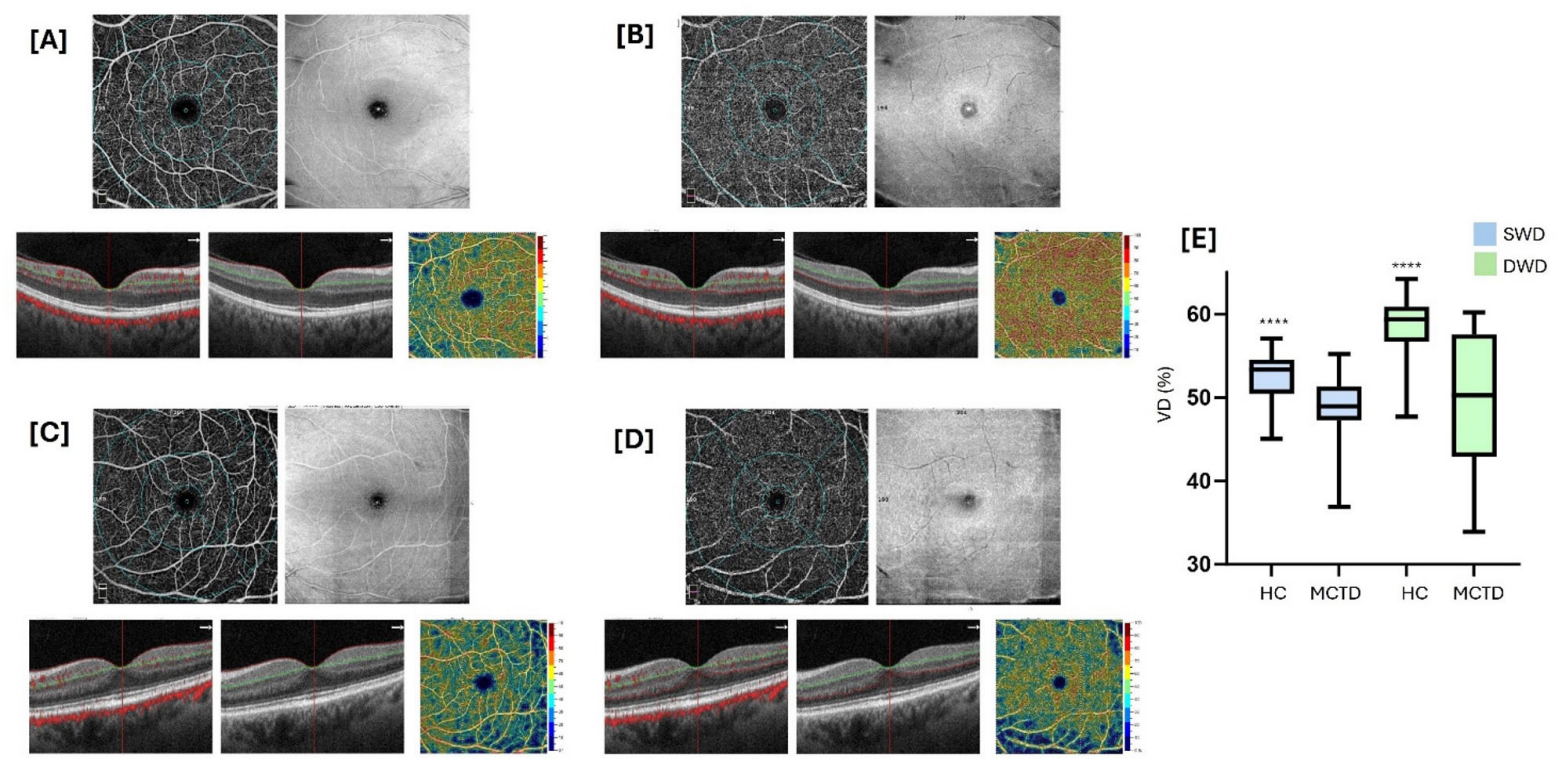
Superficial and deep retinal vessel density by optical coherence tomography angiography (OCT-A) in MCTD patients. Vessel density (VD) measures in MCTD patients and healthy controls (HC). Representative scans from OCT-A: in panels **(A, B)**, superficial and deep capillary plexi from a MCTD patient, respectively; in panels **(C, D)**, superficial and deep capillary plexi from a HC, respectively. In panel **(E)**, a boxplot describing Superficial Whole Density (SWD, in both eyes) and Deep Whole Density (DWD, in both eyes) in HC and in patients. Continuous variables were compared using the parametric unpaired T test (****P <0.0001).

Representative scans from a patient with MCTD and an HC are shown in **Figures 1A–D**. Differences in SWD and DWD between MCTD and HC groups are depicted in **Figure 1E**.

Age inversely correlated with superficial and deep retinal VD in both MCTD (SWD r = −0.4, P = 0.02; SFD r = −0.45 P = 0.004, DWD r = −0.3, P = 0.04, DFD r = −0.5, P = 0.003) and HC (SWD r = −0.3, P = 0.04; SFD r = −0.6, P <0.0001; SPFD r = −0.4, P = 0.02; DWD r = −0.6, P <0.0001; DPFD, r = −0.5, P = 0.0003). No difference in VD was observed between elderly and non-elderly subjects (data not shown).

**Figure 2:**
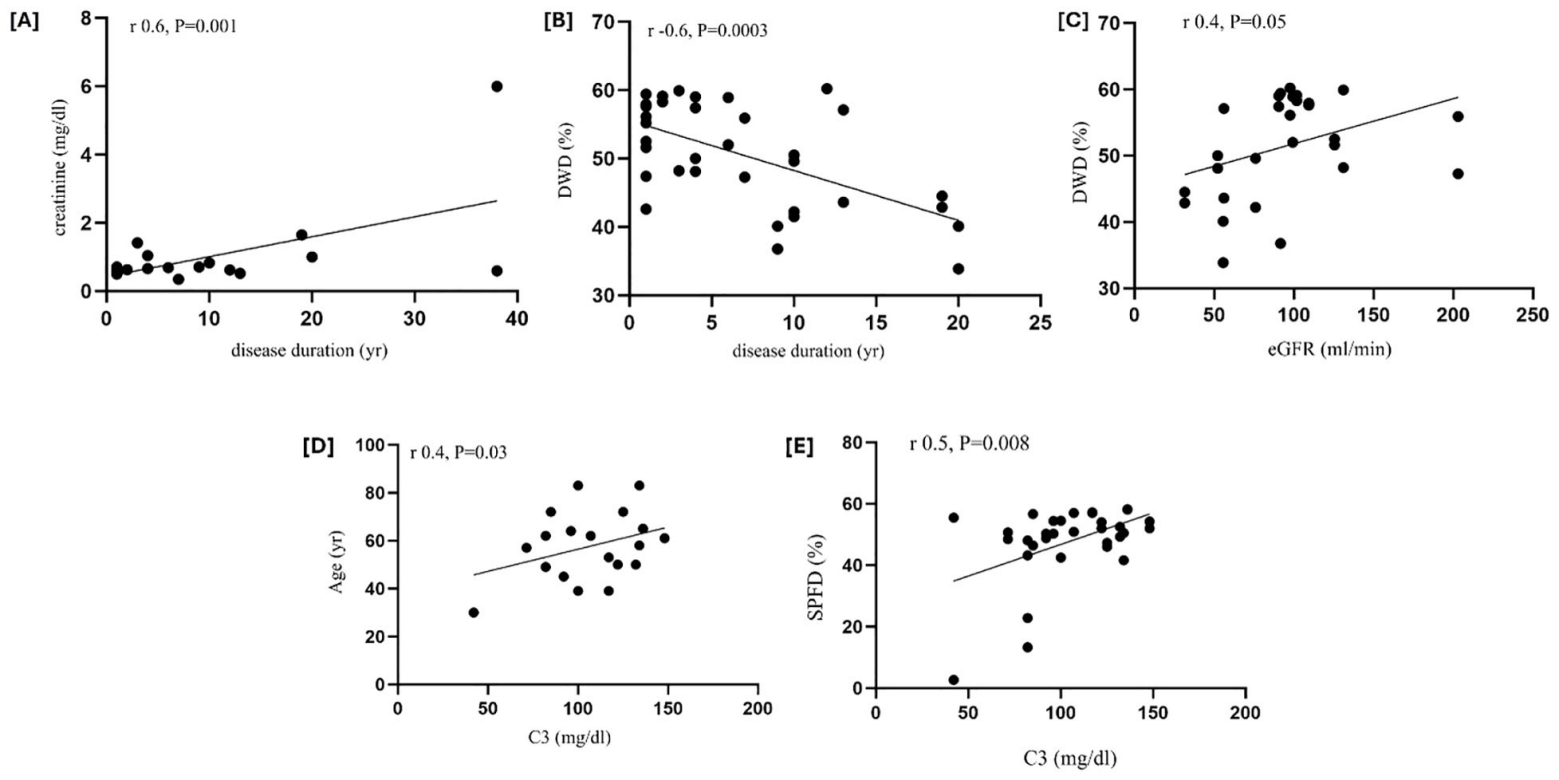
Retinal vessel density, disease duration, renal function, and C3 in MCTD patients. Disease duration correlated positively with creatinine [r = 0.6, P = 0.001, panel **(A)**] and inversely with Deep Whole Density [DWD, r = −0.6, P = 0.0003, panel **(B)**]; a positive correlation occurred between DWD and eGFR [r = 0.4, P = 0.05, panel **(C)**]; C3 significantly correlated with age of patients [r = 0.4, P = 0.03, panel **(D)**] and with superficial parafoveal density [SPFD, r = 0.5, P = 0.008, panel **(E)**]. The significance of any correlation was determined by Pearson’s correlation test.

In MCTD, disease duration was positively correlated with creatinine (r = 0.6, P = 0.001) and inversely correlated with DWD (r = −0.6, P = 0.0003, **Figures 2A, B**). Furthermore, eGFR was positively correlated with deep VD (DWD, r = 0.4, P = 0.05, **Figure 2C**). C3 was significantly related to the age of patients (r = 0.4, P = 0.03) and SPFD (r = 0.5, P = 0.008) (**Figures 2D, E**). When analyzing patients on steroids (n = 8) and those who were not on steroids (n = 12), no differences were observed in the OCT-A measurements of retinal vessel density (data not shown).

### 3.3 OCT-A measures and clinical phenotype

NVC documented non-specific microvascular abnormalities in 12/20 (60%) patients, while in fewer patients (8/20), a scleroderma-like pattern was documented. No significant correlations were observed in retinal VD according to OCT-A when comparing patients with MCTD with non-specific NVC microvascular abnormalities and those with NVC scleroderma-like patterns.

According to lung involvement, we classified 35% of the patients (n = 7) as having UIP, 20% (n = 4) with GGO, and 15% (n = 3) with NSIP. We compared OCT-A findings between patients with and without lung involvement, and no difference in OCT-A findings was observed between the two groups.

## 4 Discussion

We documented, for the first time, subclinical retinal changes in the capillary network of patients with MCTD: these microvascular changes appear to be linked with disease duration, renal function, and plasma levels of complement components. Microvascular abnormalities at NVC and lung involvement have been documented in a relevant proportion of patients with MCTD without significant correlations with retinal vessel density.

Systemic CTDs can present with a variety of clinical features, including ocular changes (10). However, retinal vascular disease has rarely been described in MCTD; reports have documented rare cases of artery occlusions and retinal vasculitis (23) that may also be refractory to immunosuppressant treatments (24, 25). In MCTD, morbidity and mortality are related to multi-organ involvement. Evidence demonstrates the heterogeneity of clinical phenotypes of MCTD and suggests that underlying vasculopathy may be a key player in tissue damage (26). In this context, the detection of retinal microvascular involvement is often a relevant diagnostic challenge leading to a worse prognosis for patients (27). Data from our pilot study suggest the utility of OCT-A as a non-invasive tool for the detection of subclinical retinal vasculopathy in patients with MCTD in whom both cardiovascular and autoimmune-mediated inflammation might lead to vascular damage. Significant correlations were observed between OCT-A abnormalities and age of the subjects, suggesting that retinal capillary plexi may represent an “aging marker” of systemic complications. However, no difference in VD was observed between elderly and non-elderly participants, supporting the idea that disease duration might play a major role in determining VD changes instead of age, even though a decrease in retinal vessel density is associated with advancing age (26). In this context, evidence reports that aging is an irreversible and multifactorial process strongly associated with oxidative stress and inflammation, which negatively affect retinal microcirculation (27). According to our findings, retinal VD appears to be linked with renal function, which, in turn, inversely correlates with MCTD disease duration. We found that disease duration was positively related to plasma creatinine and inversely related to eGFR, which directly correlated with retinal VD, thus supporting the idea that renal function can have a trend to correlate with retinal microcirculation in MCTD, as reported in other systemic chronic diseases such as CKD (28). However, this association between retinal VD and renal function (in terms of eGFR) has not been previously described in MCTD and is consistent with the link between inflammation and atherosclerotic cardiovascular diseases in CTDs (29). These original results, although they occur within normal renal ranges, might have potential clinical relevance considering that retinal microvascular abnormalities associated with systemic vasculopathy can be suggestive of renal dysfunction. Nevertheless, further prospective studies with a longitudinal design and a larger population should explore these preliminary observations and other endpoints of decline in kidney function, such as the slope of eGFR (30). In our MCTD cohort, C3 and superficial retinal VD were inversely correlated. C3 is an acute-phase protein, and elevated plasma C3 levels have been reported to be associated with the development of systemic inflammatory diseases, such as diabetes and hypertension, which are the two main causes of CKD (31). Taken together, these findings suggest that both inflammation and complement activation contribute to vascular damage in MCTD.

No differences were observed in OCT-A findings when comparing MCTD patients according to NVC patterns; no data are available in the literature on the potential link between the retinal vascular network and abnormalities on NVC in MCTD. However, patients with MCTD are significantly more likely to have NVC alterations, as recent evidence has described that scleroderma-like patterns could be found in more than half of the patients with MCTD, with potential correlations with clinical phenotype (32). In our MCTD cohort, we observed relatively low ANA titers in almost half of the cohort; this might be unexpected, but as in other CTDs, ANA titers can fluctuate according to disease activity, and low titers can suggest a good response to therapies in treated patients. Accordingly, it can be hypothesized that the relatively low rate of NVC-SSc-like patterns in our cohort could be related to the current disease control in patients undergoing chronic treatment. Notably, at the time of the study, half of the MCTD cohort was on prednisone or equivalent with a low median daily dosage. No differences have been documented in OCT-A measurements of retinal vessel density between patients according to steroid treatment; however, because of the small sample size, the statistical power to detect such differences is significantly limited. All patients in the study population underwent lung HRCT in accordance with good clinical practice at the time of the study. No difference in OCT-A values was observed between patients with and without groups. To date, no studies have explored the correlations between retinal VD measured by OCT-A and lung HRCT patterns. Further studies with larger populations are needed to obtain significant results in this context.

The main limitation of these findings is the relatively small sample size of the MCTD cohort, which does not allow adequate stratification of patients and leads to a large effect size, thus potentially overestimating the results. This is mostly due to the very low prevalence of the disease and the resulting challenges in recruiting individuals who meet the inclusion criteria and complete the study. Indeed, the identified associations should be considered preliminary observations to be further investigated in future larger studies. Correlations between retinal changes and renal function occur strictly within normal renal ranges, according to the exclusion criteria; therefore, the relevance of these correlations serves an exploratory purpose and should be considered preliminary.

Nevertheless, in this study, we analyzed retinal VD using a 6 × 6 mm OCT-A scan, which can provide a larger field of view while maintaining a high quality and details as traditional 3 × 3 mm OCT-A scans. The extent of retinal vascularization is thus visualized in a higher area captured in the 6 × 6 mm scans documenting detailed and informative data from the retinal vasculature of patients and controls (33). Therefore, we analyzed the retinal microvascular network using very accurate and innovative tools, in a non-invasive way in a selected cohort of patients affected by a rare disease.

## 5 Conclusions

Patients with MCTD show subclinical reduction in vessel density at the retinal level. This abnormal reduction in the capillary network exhibited a trend of association with disease duration, renal function, and plasma levels of C3 suggesting a potentially relevant role of OCT-A in the risk stratification of organ involvement in MCTD.

## Data Availability

The raw data supporting the conclusions of this article will be made available by the authors, without undue reservation.
